# Proteasome-associated deubiquitinases and cancer

**DOI:** 10.1007/s10555-017-9697-6

**Published:** 2017-11-14

**Authors:** Arjan Mofers, Paola Pellegrini, Stig Linder, Pádraig D’Arcy

**Affiliations:** 1 Department of Medical and Health Sciences, Linköping University, SE-581 83 Linköping, Sweden; 2 Cancer Center Karolinska, Department of Oncology and Pathology, Karolinska Institute, SE-171 76 Stockholm, Sweden

**Keywords:** Ubiquitin, Proteasome, Deubiquitinases, Cancer

## Abstract

Maintenance of protein homeostasis is a crucial process for the normal functioning of the cell. The regulated degradation of proteins is primarily facilitated by the ubiquitin proteasome system (UPS), a system of selective tagging of proteins with ubiquitin followed by proteasome-mediated proteolysis. The UPS is highly dynamic consisting of both ubiquitination and deubiquitination steps that modulate protein stabilization and degradation. Deregulation of protein stability is a common feature in the development and progression of numerous cancer types. Simultaneously, the elevated protein synthesis rate of cancer cells and consequential accumulation of misfolded proteins drives UPS addiction, thus sensitizing them to UPS inhibitors. This sensitivity along with the potential of stabilizing pro-apoptotic signaling pathways makes the proteasome an attractive clinical target for the development of novel therapies. Targeting of the catalytic 20S subunit of the proteasome is already a clinically validated strategy in multiple myeloma and other cancers. Spurred on by this success, promising novel inhibitors of the UPS have entered development, targeting the 20S as well as regulatory 19S subunit and inhibitors of deubiquitinating and ubiquitin ligase enzymes. In this review, we outline the manner in which deregulation of the UPS can cause cancer to develop, current clinical application of proteasome inhibitors, and the (pre-)clinical development of novel inhibitors of the UPS.

## The ubiquitin proteasome system (UPS)

The controlled regulation of protein turnover is crucial for the maintenance of cellular homeostasis and viability. Regulation of protein turnover occurs at multiple levels from control of gene transcription and translation to the degradation of damaged, unneeded, or short-lived proteins. Multiple checkpoints and feedback loops exist to ensure that protein homeostasis is maintained in the cell. The ubiquitin proteasome system (UPS) has emerged as an important regulator for the targeted degradation of proteins involved in diverse cellular processes such as cell cycle control, gene transcription, DNA repair, and apoptosis induction. Considering its involvement in such a broad spectrum of functions, it is not surprising that deregulation of the UPS has been shown to play a pertinent role in the development of various human diseases such as cancer, atherosclerosis/cardiovascular disease [1], and neuropathologies such as Alzheimer’s disease [2]. In this review, we focus on how deregulation of the UPS can contribute to tumorgenesis and discuss the potential of the UPS and in particular deubiquitinases (DUBs) as a promising target for the development of anti-cancer agents.

### Ubiquitination: a molecular tag that alters protein function

**Fig. 1:**
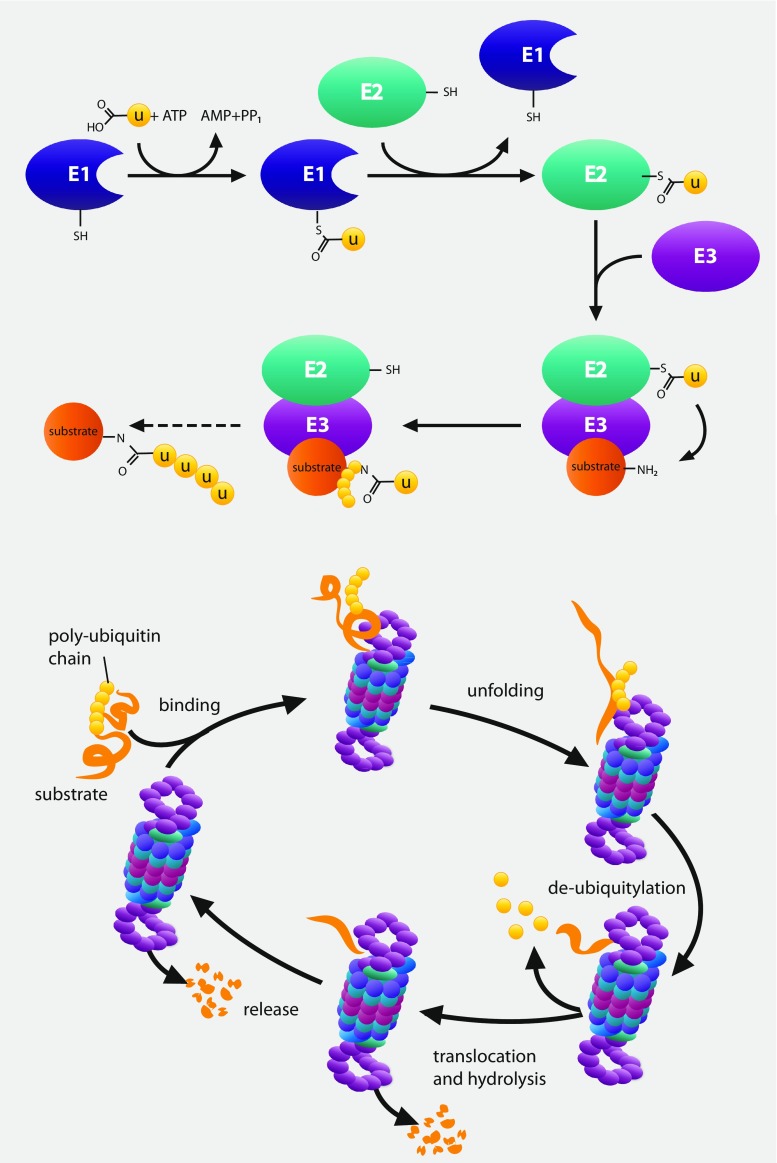
Schematic representation of the UPS. Substrate protein is tagged with ubiquitin by E1, E2, and E3. Ubiquitin-tagged proteins are recognized and degraded by the proteasome

At its simplest level, the UPS consists of a relay of enzymes that tag proteins for destruction with the small-molecule ubiquitin (Ub) and the proteasome, a highly specific molecular shredder that degrades Ub-tagged substrates (Fig. 1). The ubiquitination of proteins occurs *via* three sequential steps performed by ubiquitin-activating (E1), ubiquitin conjugation (E2), and ubiquitin ligase (E3) enzymes. The E1 enzyme initially forms a high-energy thio-ester bond with Ub in an ATP dependent manner, resulting in the activation of the Ub molecule. The active Ub forms a complex with the active cysteine in the E2 enzyme resulting in the formation of an intermediate E2-Ub complex. A compatible E3 enzyme interacts with the E2-Ub complex and target substrate, thus serving as a highly specific molecular scaffold that facilitates the conjugation of the Ub residue from the E2 to consensus lysine residues in the target protein. The number of E1, E2, and E3 enzymes is highly variably with 2 E1, over 40 E2, and several hundred varieties of E3 enzymes described [3]. A high degree of variability and target specificity exists within the E3s, with enzymes classified into three families based on sequence homology, namely the RING, HECT, or RING-between-RING ubiquitin ligases. The E3 ligases play a central role in substrate recognition and specificity in Ub conjugation. To finalize the ubiquitination process, the Ub chain attached to target proteins may be elongated by a specific E3 subtype, commonly referred to as an E4 ligase that catalyzes the formation of extended poly-Ub chains. The nature of Ub attachment whether as mono-Ub or as poly-Ub chains, composed of multiple Ub moieties, can have profound effects on a proteins activity and/or stability. Since Ub contains seven internal lysine residues, multiple conformations of linkages within the polyubiquitin chains are possible. The linkage between the Ub moieties located in the chain functions as a highly specific ubiquitin code that determines the fate of the conjugated protein. Ubiquitin linkages occurring at Lysine 11, Lysine 29, and Lysine 48 generally serves as a destruction signal for proteasomal degradation, whereas other linkage types usually regulate non-proteolytic activities that alter activity, location, or interactions of the substrate protein [4].

### Deubiquitinating enzymes (DUBs)

Ubiquitination of proteins is not a one-way process and can be reversed by a class of isopeptidases known as deubiquitinating enzymes (DUBs), which catalyze the breaking of the isopeptide bond between the C-terminal glycine of Ub and the ε-amino group of lysine residues in target proteins. In total, around 80 DUBs have thus far been identified and classified into six groups based on sequence homology of the catalytic domain: ubiquitin-specific proteases (USP), ubiquitin carboxy-terminal hydrolases (UCH), ovarian-tumor proteases [5], Machado-Joseph disease protein domain proteases (MJD), JAMM/MPN domain-associated metallopeptidases (JAMM), and monocyte chemotactic protein-induced protein (MCPIP) [6–8]. While the function of many DUBs remains to be determined, deubiquitination of mono-Ub or poly-Ub chains can induce altered protein localization, trafficking, or enhanced stability [9]. While the majority of DUBs are presumed to exist as free enzymes, several have been identified through their association of large enzyme complexes. Considering the total number of DUBs identified and the degree of variability within the different family members, they are considered to be highly druggable with various DUB inhibitors currently in various stages of pre-clinical development.

### 19S regulatory particle (19S RP)

Proteins conjugated with poly-Ub chains containing Lys-11, Lys-29, and Lys-48 linkages are generally transported to the 26S proteasome, where they are degraded by the proteolytic activities of the 20S core particle (20S CP). Entry of ubiquitinated proteins to the 20S CP is controlled by the 19S regulatory particle (19S RP), which functions as a highly specific gatekeeper allowing only those proteins displaying the correct Ub tags to enter. Poly-Ub tagged substrates are recognized by highly specific Ub receptors, Rpn10 and Rpn13 localized within the 19S RP. Once a poly-Ub protein has been captured by the Ub receptors, the AAA-ATPase subunits Rpt1–6 located at the base of the 19S RP facilitate unfolding of captured protein, gate opening of the 20S CP, and translocation into the catalytic chamber where proteolysis occurs. In addition to the 19S RP, entry into the 20S CP can also be modulated by other regulatory particles such as the 11S regulator (also referred to as PA28) that replaces the 19S RP on the 20S CP and forms the immunoproteasome. While not as extensively characterized as the conventional 26S proteasome, studies have shown that immunoproteasome is involved in diverse functions, such as antigen processing and modulating the response to oxidative stress. However, the role of the immunoproteasome is outside the scope of this review. One particular aspect of the 19S RP is how polyubiquitin chains are removed from target proteins following capture by the proteasome. The presence of large, bulky poly-Ub chains poses a potential problem for proteasomal degradation, since polyubiquitinated proteins can have molecular weights much greater than non-ubiquitinated species. In order to facilitate unfolding and translocation through the narrow gate into to the 20S CP, the poly-Ub chain must be removed from the target protein. This process of proteasome deubiquitination is mediated by the action of 3 DUBs, POH1/Rpn11, UCHL5/UCH37, and USP14 [10–14].

### 20S core particle (20S CP)

The 20S core subunit is a cylindrical structure comprised of two outer heptameric α subunits stacked on two central heptameric β subunits [15, 16]. The α ring forms a physical barrier that protects against promiscuous entry to the proteolytic chamber formed by the β subunits. The catalytic activity of the 20S CP is exerted by β1, β2, and β5 subunits responsible for caspase-like, trypsin-like, and chymotrypsin-like activities, respectively [17]. The cumulative effect of these different activities results in the degradation of protein substrates into short oligopeptides generally between 3 and 15 amino acids in length. Oligopeptides are further hydrolyzed to single amino acid residues by oligopeptidases located in the cytosol, thus facilitating the recycling of amino acids for protein synthesis.

## The UPS in cancer

Deregulation of the UPS has been reported in numerous types of cancer [18, 19]. Some of the main players of the UPS and the mechanisms, by which they are postulated to drive cancer formation, are summarized in Table 1. Considering the broad range of UPS regulators and the cancer pathways they mediate, it is of no surprise that the UPS represents a potential treasure trove of drug targets for the development of future anti-cancer therapies.

**Table 1:** Summary of UPS subunits involved in formation of cancer. Where known, specific cancer subtypes in which the alteration is involved are indicated [17, 18]

UPS subunit	Mutation or deregulation	Malignancy	References
E3	HDM2	Various	[19–24]
	Overexpression, loss of tumor suppressor function through p53		
	FBW7	Leukemia, cholangiocarcinoma, gastrointestinal, and endometrial cancer	[25–34]
	Mutant, loss of tumor suppressor function through cyclin E, MYC, JUN, and Notch		
	SKP2	Colorectal, breast, biliary tract, and prostate cancer. NSCLC	[35–42]
	Mutant, loss of tumor suppressor function through p27		
	VHL	Lung cancer, clear-cell carcinoma, VHL disease	[43]
	Mutant, loss of tumor suppressor function through HIF		
DUB	USP1	Fanconi anemia (leukemia risk factor)	[44]
	Mutations in FANCD2 DNA repair pathways		
	USP2	Prostate cancer	[45]
	Stabilizes HDM2, facilitates malignant metabolic profile through fatty acid synthetase activation		
	USP4	Adenocarcinoma, breast cancer	[46, 47]
	Interactions with retinoblastoma protein, SMAD4, and β-catenin		
	USP8		[48]
	Regulates expression of EGFR		
	USP9x	Leukemia, myeloma, lymphoma, and pancreatic cancer	[49–51]
	Stabilizes β-catenin, SMAD4, and BCL1 family protein MCL1		
	USP15	Glioblastoma	[52]
	Stabilizes SMAD4		
	USP18	Leukemia	[53]
	USP19		[54]
	Stabilize anti-apoptotic regulators c-IAP1 and c-IAP2		
	USP28		[55]
	Stabilizes c-MYC		
	USP7, USP2a, USP10	Prostate cancer	[56, 57]
	Stabilize p53		
19S	USP14	Lung adenocarcinoma	[58, 59]
	Stabilization of various regulators including IκB and β-catenin		
	POH1/Rpn11		[60]
	Stabilization of c-JUN		
Other	Human papilloma virus onco-protein	Cervical, head-and-neck cancer	[61, 62]
	Viral particle that mimics E3 activity, altering stability of various substrates including p53 and MYC		

### Targeting the UPS in cancer

The manner by which proteasome inhibition induces apoptosis in cancer cells is not fully understood. Since the UPS is involved in such a broad range of processes, the manner in which apoptosis is induced is at least in part context dependent. Four potential mechanisms by which proteasome inhibition induces apoptosis and cell death are discussed below.

#### Proteotoxic stress

**Fig. 2:**
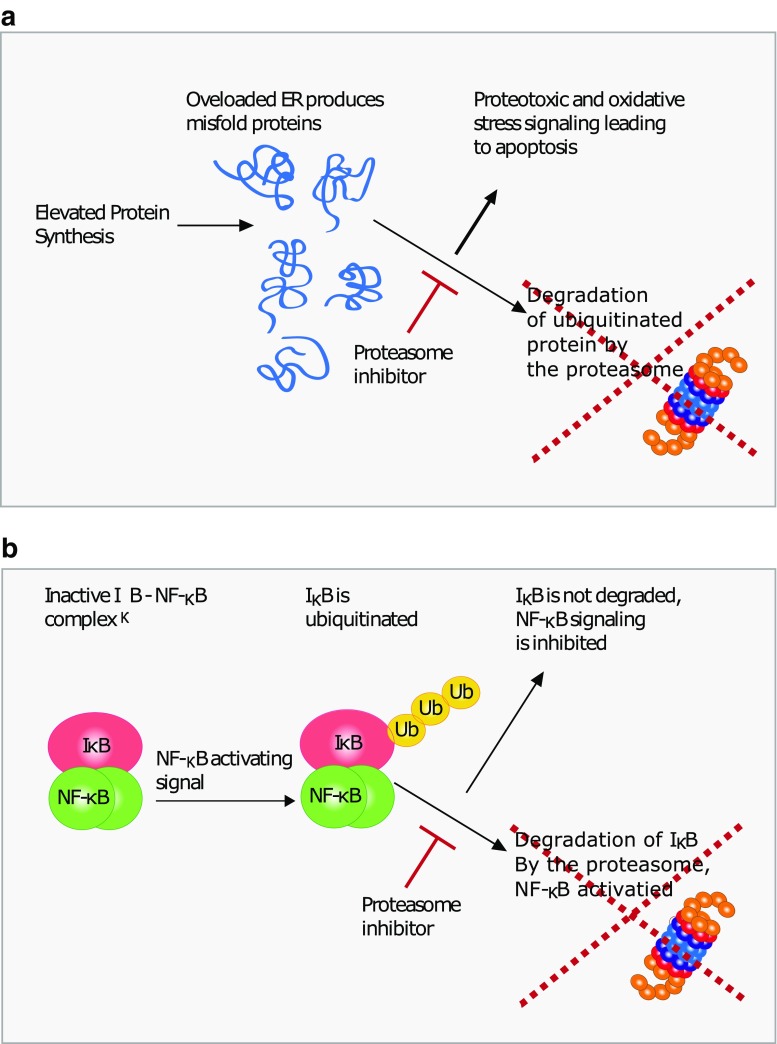
Mechanisms of how proteasome inhibition inhibits cancer cell survival. **a** Generation of misfolded proteins due to elevated protein synthesis in cancer cells requires the UPS for detoxification. Proteasome inhibition leads to accumulation of misfolded proteins, causing proteotoxic and oxidative stress. **b** Proteasome inhibition leads to abrogation of NFκB pro-survival signaling

Malignant neoplastic cells are generally characterized as having significantly elevated protein synthesis rates, often due to alterations in mTOR signaling or growth signaling pathways [20]. Excessive protein synthesis overloads the ability of the endoplasmic reticulum (ER) to mediate protein folding, leading to acute proteotoxic stress (Fig. 2A). Since the UPS is the primary system responsible for the degradation of misfolded proteins, it is tempting to argue that cancer cells are particularly sensitive to proteasome inhibition when compared to healthy cells due in part to enhanced rates of misfolding that occur as a consequence of excessive protein translation. ER stress is a potent signal for apoptosis, where the accumulation and aggregation of misfolded proteins lead to disruption of membranes and normal cellular functions [21]. Consistent with this hypothesis, we and others have shown that an acute ER stress response followed by apoptosis sequentially occurs following treatment with various proteasome inhibitors.

#### Oxidative stress

Protein oxidation is a necessary step in protein folding where the oxidation of disulfide bonds is facilitated by chaperones localized in the ER [22]. However, proteins are also susceptible to oxidative modifications as a consequence of oxidative stress. Proteasome inhibition is associated with the induction of an acute oxidative stress response that appears to be the driving factor leading to apoptosis in various cancer models (Fig. 2A) [23–27]. While the exact connection between oxidative stress and proteasome inhibition is far from clear, the accumulation of misfolded proteins followed by unsuccessful attempts of protein refolding by the cells’ chaperone system is a likely culprit. Of particular interest is the finding that attenuation of ROS generation by anti-oxidants significantly reduces proteasome inhibitor induced apoptosis, indicating that oxidative stress plays a major role in apoptosis induction following UPS inhibition [23, 26].

#### Targeting signaling pathways

Initially, the rational for proteasome inhibitors as a potential for cancer treatment was the disruption of pro-survival signaling by UPS regulated transcription factors. IκB, which functions as an inhibitor of the NF-κB transcription activator complex, represents such a target (Fig. 2B). Proteasome inhibition leads to the rapid accumulation of IκB, which in turn associates with NF-κB and blocks the activation of downstream signaling. However; this does not appear to be a universal effect, since studies have shown that inhibition of IκB degradation and activation of NF-κB can occur simultaneously in cells treated with bortezomib [28]. For a more in-depth review regarding NF-κB and proteasome inhibition, we refer to Bedford [29].

In addition to NF-κB, regulators of the cell cycle have also emerged as targets of UPS inhibition. The cyclins are a broad class of cell cycle regulators under the control of the UPS. The cell cycle is strictly regulated to ensure that external mitogen signaling is coupled to cell division. This process is tightly controlled by a cohort of both positive (cyclins and cyclin-dependent kinases (CDK)) and negative (CDK inhibitors) regulators that facilitate timely progression through the cell cycle phases. Cyclins are very short-lived regulators of CDK activity and are subject to degradation by the proteasome in a cell cycle phase-specific manner [30]. Considering that numerous cell cycle regulators are UPS substrates, it is of no surprise that proteasome inhibition results in deregulated cell cycle progression and growth arrest in the G1 and G2 phases of the cell cycle.

#### DNA repair

Double-stranded DNA breaks (DSB) occur naturally during cell division, either *via* defects in replication or through exposure to DNA damaging agents. The deregulation of cell growth that occurs in cancer increases the frequency of DSBs, where increased genomic instability can further contribute to cancer progression by activating mutations in proto-oncogenes [31]. Crucial to induction of apoptosis in response to DSBs is the tumor suppressor p53. p53 levels are regulated by the E3 enzyme HDM2, which ubiquitinates p53, thus targeting it for proteasome-mediated degradation. p53 and HDM2 form a negative feedback loop, where increasing p53 levels activate HDM2 transcription; however, in cases of DNA damage, the p53-HDM2 circuit is disrupted leading to stabilization of p53 tumor suppressive function. Activating mutations or amplification of the HDM2 gene is observed in numerous tumor types resulting in enhanced p53 ubiquitination and proteasomal degradation. Rescue of p53 function inhibiting its degradation has been shown to be a viable anti-tumor strategy with several inhibitors of the HDM2-p53 circuit in various stages of clinical trials [32, 33].

#### Specific degradation of onco-proteins

The targeted inhibition of onco-protein activity is a major challenge. Certain classes of onco-proteins can be inhibited by compounds that block their active site; however, not all onco-proteins are “druggable” in this manner. Inhibition at the transcription or translation level is similarly limited, with miRNA therapy still in the early stages of clinical development. Exploiting the UPS to specifically degrade onco-proteins is an emerging method of treatment. Increased protein degradation has been observed as a side effect of some inhibitory small-molecule drugs, largely through un-elucidated mechanisms. Examples of onco-proteins targeted by such “double effect” small molecules include HER2/neu and estrogen receptor α, both important drivers in cancer [34, 35]. Further advancements have been made with proteolysis targeting chimera (PROTAC) technology. PROTACs are molecules containing a recognition site for the target protein as well as a recruiter region for an E3 ligases. This forces ubiquitination of the target protein, even if the protein is not usually a substrate for that particular E3 ligase. The technique has already shown to be capable of causing selective degradation of certain onco-proteins, and further development is expected to increase both the amount of viable targets and the pharmacokinetics of PROTACs [36].

## Multiple myeloma and mantle cell lymphoma: promising targets for proteasome inhibitor-based cancer treatment

At first glance, the rational for proteasome inhibition as treatment for cancer appears simple, namely, the deregulated UPS activity that occurs can contribute to tumorgenesis *via* the deregulation of factors involved in proliferation and apoptosis. However, clinical experience has shown that certain cancer types are more susceptible to proteasome-based treatments than others. In the current clinical practice, proteasome inhibitors are used to treat multiple myeloma and mantle cell lymphoma (MCL) [37], with clinical trials currently underway for non-small cell lung cancer (NSCLC) [38, 39].

The potential of proteasome inhibitors as an anti-cancer therapy was effectively shown by the success of bortezomib as a treatment option for MM. MM is a cancer of the antibody producing plasma B cells that typically reside in the lymphatic tissues. The microenvironment composed of tumor and healthy stromal marrow cells provides for a self-perpetuating loop of cytokine release that sustains MM proliferation and dampens the apoptotic response [40]. Multiple mechanisms have been proposed to explain the anti-tumor effect of bortezomib. Downregulation of the NF-κB pathway following bortezomib treatment has been well described in MM models; however, this mechanism alone is insufficient to explain the efficacy of bortezomib. Since MM displays hyperactive protein synthesis rates due to high levels of antibody secretion, such cells can be thought of as being primed for ER stress-induced apoptosis. Consistent with this, expression levels of IgG and the corresponding level of ER stress are shown to be a strong predictor of bortezomib toxicity [41].

MCL is a relatively rare B cell non-Hodgkin lymphoma, representing 3–6% of all non-Hodgkin lymphomas. The disease is highly aggressive and in the majority of cases, patients eventually relapse. As such, there is no established standard of care for patients who cannot tolerate high-intensity treatment [42]. A typical feature of MCL is the presence of the reciprocal chromosome translocation, t(11;14)(q13;q32), that is the presumed initial driving factor. The translocation places the proto-oncogene CCND1, encoding for cyclin D1, under the transcription regulation of the immunoglobin heavy chain locus. The constitutive expression of cyclin D1 results in the aberrant activation of CDK4/6 and the uncoupling of cell cycle regulation from external mitogenic signaling. The occurrence of additional mutations in oncogenes and tumor suppressor genes also contributes to the aggressive nature of MCL, reviewed in detail by Jares [43]. Disease progression occurs in the lymph node mantle region, where MCL cells accumulate and form tumors and subsequently spread through the lymph system, blood, or to adjacent tissue. Similar to MM, bortezomib has received approval by the FDA and EMA for the treatment of MCL.

Although proteasome inhibitors have shown good activity in hematological malignancies such as MM and MCL, a relatively limited success has been observed for the treatment of solid tumors. One possible explanation is that the pharmacokinetics and pharmacodynamics of these compounds improve bioavailability in the blood and bone marrow environment compared to solid tumors. To circumvent this, the use of higher doses has been proposed; however, unwanted side effects and toxicity generally limit dosage. Considering these limitations, there has been increased interest in the search for the next generation of UPS inhibitors with better bioavailability and activity on a broader range of tumor types.

## 20S proteasome inhibitors

20S proteasome inhibitors are classified in three categories: boronates, including bortezomib and ixazomib; epoxyketones such as carfilzomib and oprozomib; and finally salinosporamides such as marizomib. Here, we summarize the clinical aspect of 20S proteasome inhibitors that are either approved or at advanced stages of clinical trials. A brief overview of proteasome inhibitors is shown in Table 2.

**Table 2:**
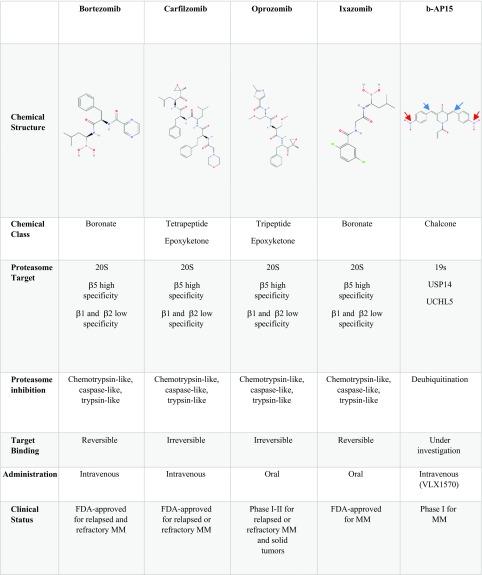
Summary of proteasome inhibitors in clinical use or early-stage clinical development

### Bortezomib

Bortezomib (PS-341, NSC 681239, Velcade®; Millennium Pharmaceuticals, Inc.) was the first proteasome inhibitor approved by the U.S. Food and Drug Administration (FDA) for the treatment of relapsed MM [44] and MCL [45]. Bortezomib was initially used in the clinic as single agent for the treatment of MM patients who relapsed after standard dexamethasone therapy, where it resulted in an improved overall survival rate. Subsequent studies have shown that bortezomib treatment for newly diagnosed MM significantly increased patient survival rates [46].

Bortezomib is a synthetic dipeptide boronic acid, which slowly and reversibly inhibits the chymotrypsin-like and to a lesser extent the trypsin-like and caspase-like activities of the 20S CP. Inhibition is achieved through an initial nucleophilic attack on the active N-terminus threonine residues located on the β5, β2, and β1 subunits, leading to loss of proteolytic activity [47]. It has been reported that bortezomib inhibits the activation of NF-κB pathway in squamous cell carcinoma [48], MM [49]and in MCL [50]. However, other studies have reported that canonical NF-κB activation is induced by bortezomib in MM cells suggesting that its cytotoxic effects cannot be entirely attributed to inhibition of NF-κb [28]. For this, the anti-cancer and pro-apoptotic activities of bortezomib may cell type-dependent and driven by specific signaling pathways. It has been reported that bortezomib induces both transcriptional and post-translational increases in cyclin-dependent kinases, p21 and p27 in hepatocellular carcinoma cells, and NSCLC [51, 52]. Bortezomib-mediated proteasome inhibition is p53-dependent in renal and NSCLC cell lines [52, 53]. Bortezomib activates the stress kinase JNK and the c-Jun/AP-1 signal pathway, thus inducing the expression of the cyclin-CDK inhibitor p21, leading to growth arrest and apoptosis of NSCLC cells [52]. Finally, the induction of pro-apoptotic protein NOXA by bortezomib is directly dependent on the oncogene MYC in melanoma cell lines [54].

Despite promising results in MM patients, several limitations of bortezomib treatment have emerged. The lack of response observed in some patients as a first-line therapy and relapses and/or resistance after initial favorable responses in MM patients have been described as the main limitations of the drug [55]. Moreover, bortezomib treatment has been correlated to the onset of adverse effects such as thrombocytopenia, fatigue, GI symptoms, and asthenia. Bortezomib-induced peripheral neuropathy (BIPN) was identified as the main significant dose-limiting toxicity potentially leading to permanent nerve damage to extremities probably due to an off-target effect of the drug [56] . The incidence of BIPN was reduced by changing the dose frequency from twice weekly to once weekly and choosing subcutaneous administration over intravenous injection [57–61]. In addition, bortezomib has shown successful results when used in combination with other type of treatments. Combination treatments of bortezomib with doxorubicin, dexamethasone, panobinostat, and daratumumab showed improved clinical outcomes in patients with relapsed and refractory MM, suggesting its ability to sensitize malignant cells to conventional chemotherapy [61–63]. However, less promising results were obtained in patients with solid tumor compare to those with hematologic malignancies. The combinational treatment of bortezomib with docetaxel in prostate tumor did not reveal any significant anti-cancer effect [64].

Several mechanisms of resistance have been reported and investigated in pre-clinical and clinical settings. Alterations at the proteasome level include mutations and overexpression of the catalytic subunits. For instance, alteration of the gene *PSMB5* encoding for the proteasome subunit β5 was found in tumors resistant to bortezomib *in vitro* and in pre-clinical studies [65–67]. However, no such mutations were found in MM patients who developed resistance to bortezomib [68]. Resistance to bortezomib downstream of the proteasome includes enhanced activation of the aggresome-autophagy pathway, alterations in apoptotic signaling, increased expression of anti-oxidants, and dampening of the ER stress response. The role of the aggresome-autophagy pathway has been investigated as a resistance mechanism exploited by cells to overcome proteotoxicity induced by proteasome inhibition. It has been shown that pancreatic cancer cells treated with bortezomib form aggresomes, which are large aggregates of Ub-conjugated proteins that are subsequently cleared by autophagy. Studies revealed that inhibition of aggresome formation with histone deacetylase inhibitors such as panobinostat increases bortezomib anti-cancer activity, suggesting that aggresome formation may be a potential resistance mechanism [69, 70]. Anti-apoptotic Bcl-2 mediators have also found to be overexpressed in bortezomib-resistant cells, also implicating a reduction in apoptotic capacity as a potential resistance mechanism [71, 72]. Consistent with this, it has been shown that combination treatments with inhibitors of Bcl-2 and bortezomib showed a synergistic effect [71, 73]. Overexpression of the chaperone BIP/Grp78 protein involved in the ER stress response to proteotoxicity has also been associated with a reduced sensitivity to bortezomib, which is not surprising considering the role of ER stress in triggering apoptosis. Pretreatment of bortezomib-resistant cells with inhibitors of ER-mediated protein folding restored cell sensitivity to bortezomib, implying that enhanced chaperone activity could also attribute to bortezomib resistance [74].

### Carfilzomib

Carfilzomib (PR-171; Kyprolis; Onyx Pharmaceutical) is a tetrapeptide epoxyketone, selective and irreversible inhibitor of the chymotrypsin-like activity of the proteasome. Carfilzomib was approved by the FDA in 2012 for the treatment of relapsed MM patients who had previously received at least two therapies, including bortezomib and immunomodulatory drugs, and displayed disease progression within 60 days after the first cycle of therapy [75]. It is used as single agent in third-line treatments and in combination with lenalidomide and dexamethasone as second-line therapy [76].

Carfilzomib covalently irreversibly inhibits the β5 subunit, responsible for the chymotrypsin-like activity of the 20S proteasome, resulting in a similar stress response to that observed following bortezomib [77, 78].

Carfilzomib displayed higher cytotoxicity than bortezomib in several cell lines derived from hematologic tumors as well as solid cancer [76]. The explanation is found in the higher selectivity of the epoxyketone for the N-terminal threonine active site of the proteasome compared to the boronic acids of bortezomib and β-lactone of salinosporamide A [78, 79]. Carfilzomib is considered the most specific and potent proteasome inhibitor with good cytotoxic activity in bortezomib-resistant MM cell lines and in samples from patients with bortezomib-resistant MM [80].

To date, most of the clinical trials with carfilzomib have been performed in patients with relapsed MM. However, many other studies are ongoing and/or recruiting patients with other hematological diseases, such as Hodgkin lymphoma or solid tumors like ovarian and kidney cancer. The treatment design consists of the use of carfilzomib as a single agent or in combination with conventional treatments, such as dexamethasone, melphalan, panobinostat, and irinotecan. The new phase III CLARION study is recruiting patients with newly diagnosed MM to treat with carfilzomib or bortezomib in combination with conventional drugs to evaluate if carfilzomib can replace bortezomib as front line treatment for MM [81].

Carfilzomib is associated with several side effects mainly correlated not only to the cardiovascular system, such as hypertension, but also to the urinary tract. Moreover, carfilzomib can lead to the onset of fever, anemia, diarrhea, fatigue, and nausea [82, 83]. The main adverse effects of carfilzomib differ from bortezomib suggesting a potential difference in downstream effects of the two drugs [84].

### Oprozomib

Oprozomib (ONX0912; PR-047), a truncated derivate of carfilzomib, is a tripeptide epoxyketone that functions as an irreversible and selective inhibitor of the chymotrypsin-like activity 20S CP. The need to find better proteasome inhibitor with better dose flexibility and convenience for patients led to the design and synthesis of oprozomib as a new generation of proteasome inhibitor with better oral bioavailability compared to the intravenously administrated carfilzomib [85].

Oprozomib has shown similar anti-tumor activity, potency, and selectivity as carfilzomib on MM cell derived from relapsed patients after treatment with conventional anti-MM drugs and therefore can be used to treat patience with resistance to bortezomib, dexamethasone, or lenalidomide. *In vitro* studies showed that oprozomib has synergistic/additive anti-MM activity when combined with bortezomib, lenalidomide, and dexamethasone and inhibits the migration of MM cells and angiogenesis. Moreover, oprozomib inhibits the tumor growth in human MM xenograft models reducing tumor progression and increasing the survival [86]. The inhibitory activity of oprozomib in pre-clinical models of solid tumors had also been investigated. Results showed that oprozomib induced apoptotic pathway through Bik upregulation and activation of caspase-8, caspase-9, caspase-3, and PARP cleavage in head and neck squamous cell carcinoma (HNSCC) cells, leading to cell death. This effect is antagonized by upregulation of Mcl-1 and anti-apoptotic Bcl2 family member that inhibits cytochrome C release form the mitochondria. In response to proteasome inhibition, HNSCC cells upregulate autophagy and ATF4 resulting in increased cell survival. Oprozomib has shown promising inhibition of tumor growth in HNSCC xenograft mice models providing the bases for a further use of this drug in the clinic [87]. Oprozomib also inhibits NF-κB and activates the JNK pathways [88]. Oprozomib, similar to carfilzomib, not only targets myeloma growth directly but also decreases myeloma-associated bone disease by inhibition of osteoclast differentiation and reabsorption while enhancing osteoblast formation and function [89]. Oprozomib is in phase I/II clinical trial for treatment of newly diagnosed, relapsed, and refractory MM as a single agent or in combination with other anti-MM drugs such as dexamethasone. A phase I study in solid tumors has also been performed; however, gastrointestinal toxicities and minimal anti-tumor activity in patients with advanced solid tumors were reported [90].

### Ixazomib

Ixazomib citrate (MLN9708, Ninlaro®, Takeda Pharmaceutical, Cambridge, MA, USA) and its biologically active form ixazomib (MLN2238) are the first orally administrated proteasome inhibitors tested in the clinic for the treatment of relapsed and refractory MM [91, 92].

Ixazomib, like bortezomib, is a dipeptide boronate that reversibly and selectively targets the β5 proteasome subunit inhibiting the chymotrypsin-like proteasome activity with IC_50_ value of 3.4 nmol/L. Moreover, at higher doses, it also inhibits the β1 caspase-like and β2 trypsin-like proteolytic subunits with an IC_50_ of 31 and 3500 nmol/L, respectively [93].

Ixazomib differs from bortezomib in its physiochemical properties leading to improved pharmacokinetics and pharmacodynamics. In fact, although both drugs have a similar potency and selectivity, ixazomib has a shorter proteasome dissociation half-life (18 min *vs.* 110 min). This correlated to an improved blood and tissue distribution, which make administration of higher doses possible. Moreover, increased expression of biomarkers of proteasome inhibition, such as ER stress markers in xenograft tumor tissue, suggests improved pharmacodynamic properties. Improved pharmacokinetic and pharmacodynamic profiles have been correlated to the increased anti-cancer activity of ixazomib compared with bortezomib. In fact, ixazomib showed a better anti-cancer activity in pre-clinical studies performed on solid tumor and hematologic xenograft mice models [93, 94].

Ixazomib also showed synergistic anti-MM activity when used in combination with dexamethasone or lenalidomide [93, 95].

A study on microRNA profiling of MM cells treated with ixazomib showed upregulation of the small long-coding RNA miR33b, which seems to be constitutively under-expressed in MM patients. Overexpression of miR33b led to tumor growth inhibition and increased survival in human MM xenograft mice model, elucidating its tumor suppressor role during apoptosis induced by ixazomib treatment [96].

Ixazomib was approved in 2015 in the USA by FDA and in 2016 in EU by EMA. Ongoing clinical trials are investigating its activity, as single agent or in combination with other standard anti-MM drugs, like melphalan, prednisone, lenalinomide, and dexamethasone, in hematological and solid tumors. Results have shown an evident anti-MM activity and good tolerability and safety [97]. Like bortezomib, ixazomib’s most common side effects are thrombocytopenia, gastrointestinal symptoms, neutropenia, and fatigue but with a lower incidence of neuropathy, probably due to its higher selectivity [37].

## DUB inhibitors: a way to overcome 20S inhibitor resistance?

Despite the success of bortezomib and the next generation of proteasome inhibitors in the treatment of MM and MCL, acquired resistance and progression to relapse are unfortunately a common event. A tempting alternative to the targeting the proteolytic activities of the proteasome is to target upstream regulators of the UPS such as components of the Ub conjugating or DUB machinery. In this section, we will focus on the potential of targeting DUBs, with particular emphasis on the proteasome-associated DUB that modulates deubiquitination at the proteasome (Fig. 1). The cysteine residues in the active sites of many DUBs potentially represent a blessing and a curse. While cysteine residues are considered highly druggable, cysteines are widespread in the proteome and are generally nucleophilic and thus susceptible to electrophile “attack.” Naturally, this brings into question the specificity of small-molecule compounds designed to target specific cysteines. Investigation so far, however, shows modest off-target activity for DUB inhibitors, or at least has limited reactivity outside of the DUB family. The likely cause for this is that while these electrophilic compounds can theoretically attack many cysteines, their relatively low electrophilicity appears insufficient to target a wide range of cysteines. So, while cysteines are abundant in the proteome, it appears that their configuration in the active sites of DUBs makes them particularly attractive targets for electrophilic inhibitors.

### b-AP15 and VLX1570

**Fig. 3:**
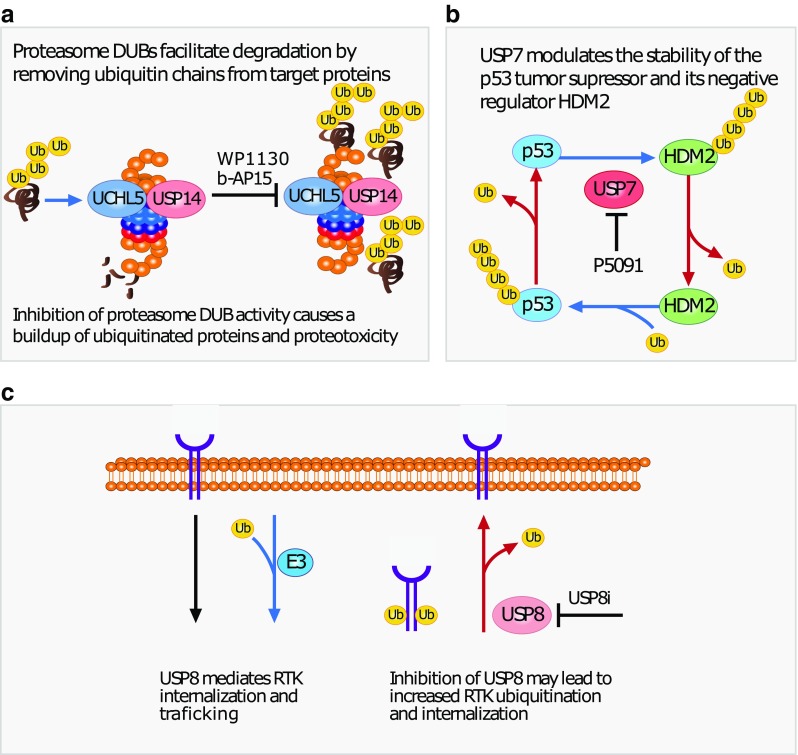
Summary of DUB inhibitors and their effect. **a** b-AP15 and WP1130 inhibit proteasomal DUBs, preventing deubiquitination at the proteasome. Blocking of deubiquitination prevents the proteasome from processing ubiquitin-tagged proteins. **b** P5091 inhibits USP7. Inhibition of USP7 prevents deubiquitination of HDM2, destabilizing it, which in turns stabilizes p53. p53 activates pro-apoptotic pathways, causing tumor cell death. **c** USP8i inhibits USP8. USP8 mediates the recycling of RTKs from the cell surface. Inhibition by USP8i may reduce surface expressed levels of RTKs, providing an alternative avenue of inhibiting oncogenic RTK signaling in EGFR inhibitor-resistant tumors

b-AP15 (3E,5E-bis[(4-nitrophenyl)methylene]-1-(1-oxo-2-propen-1-yl)-4-piperidinone) (Fig. 3A) is a small-molecule inhibitor of proteasomal DUBs USP14 and UCHL5 [98]. Its predicted method of action is inhibition of these proteasomal DUBs, blocking the deubiquitination process. The b-AP15 molecule, and its optimized lead VLX1570, contains two α,β-unsaturated carbonyl groups (Table 2, indicated by blue arrow) which, together with electron drawing capacity from side groups (Table 2, indicated by red arrow), can facilitate Michael addition to the thiols of cysteine residues of the DUB active sites.

In pre-clinical models, b-AP15 shows to be potent in causing accumulation of ubiquitinated proteins without affecting the activity of the 20S [23, 99], consistent with the idea that proteasomal DUB inhibition selectively causes a block in the deubiquitination and subsequent degradation of proteins by the proteasome. b-AP15 response is characterized by strong induction of HMOX-1 indicating a crucial role for oxidative stress in apoptosis induced by b-AP15 [23]. As of yet, it is unclear whether this induction of HMOX-1 is caused as a direct consequence of accumulation of ubiquitinated proteins, or whether off-target activity is the cause. While some off-target activity of b-AP15 has been determined, in the form of inhibition of thioredoxin reductase (TrxR), TrxR inhibition by itself accounted for neither the ubiquitinated protein accumulation nor the induction of apoptosis [23]. Of particular interest in the context of overcoming bortezomib resistance is the finding that b-AP15-induced apoptosis in bortezomib-resistant MM cells. Cells resistant to bortezomib show marked induction of anti-apoptotic regulator BCL2 and reduction of pro-apoptotic proteins Bax and Bak [23, 98, 100]. While sufficient to perturb apoptosis induced by bortezomib, bortezomib-resistant cells maintained their sensitivity to b-AP15. This indicates that b-AP15 induces apoptosis by a distinct pathway from bortezomib suggesting a potential treatment for patients with acquired bortezomib resistance.

Resistance to b-AP15 itself in clinical setting is likely inevitable, but pre-clinical assessment of acquired resistance to b-AP15 shows promising results. After 9 months of continues low-grade exposure to b-AP15, cells only became ~ twofold more resistant [101]. Mutations to the active site of USP14 or UCHL5 that would silence inhibition by b-AP15 are unlikely to be functional and to the best of our knowledge have not been observed. Upregulation of detoxification enzyme glutathione is another likely method by which resistance could be induced, but screening of cell lines with known levels of glutathione showed no correlation between these levels and the ability of b-AP15 to induce apoptosis [102].

### WP1130 and EOAI3402143

WP1130 (degrasyn, (2E)-3-(6-bromo-2-pyridinyl)-2-cyano-N-[1S-phenylbutyl]-2-propenamide) (Fig. 3A) is a partially selective DUB inhibitor initially identified as a stabilizer of Bcr/Abl in chronic myelogenous leukemia [103] and a negative regulator of MYC in melanoma [104]. WP1130 inhibited tumor growth *in vivo* at doses that were well tolerated. Further studies in MM identified the compound as an inhibitor of USP9x [5], a DUB necessary for stabilizing the anti-apoptotic BCL2 family protein MCL1 [105]. In response to WP1130 treatment, USP24 was upregulated and acted as a compensatory mechanism for USP9x inhibition by WP1130. Consequently, stability of MCL1 was eventually reestablished after WP1130 treatment. Within the same study, however, the novel WP1130 analogue EOAI3402143 was able to inhibit both USP9x and USP24 suggesting improved clinical potential [5].

### P5091

P5091 (1-[5-[(2,3-dichlorophenyl)thio]-4-nitro-2-thienyl]-ethanone; 1-[5-(2,3-dichlorophenyl)sulfanyl-4-nitro-2-thienyl]ethanone) (Fig. 3B) was first identified as a specific inhibitor of USP7 [106]. The compound was shown to inhibit stabilization of HDM2 by USP7, exerting its pro-apoptotic effect through the resulting stabilization of p21 and p53. Interestingly, p53 negative cells were also sensitive to P5091 suggesting p53 independent mechanisms [106]. A remarkable inhibition of angiogenic markers and synthesis of tumor vasculature in xenograft models was observed, contributing to its anti-tumor effect. P5091 has been studied extensively in recent times, and various new studies have shown the efficacy of the compound in a range of different cancer types and through different mechanisms such as destabilization of DNA repair regulator CCDC6 in lung-neuroendocrine cancer in conjunction with PARP inhibitors [107] and prostate cancer [108], destabilization of β-catenin in colorectal cancer [109], and activation of the p53/p21 signaling axis in chronic lymphocytic leukemia [110]. P5091 further showed strong synergy in combination treatment with RRx-001 (1-bromoacetyl-3,3-dinitroazetidine) in MM [111]. RRx-001 selectively induces oxidative and nitrative stress in hypoxic environments [112, 113], acting as a potent inhibitor of tumor growth, invasiveness, angiogenesis, and overcoming resistance to various MM treatment modalities including bortezomib.

### USP8i

USP8i (9-ethyloxyimino-9H-indeno[1,2-b]pyrazine-2,3-dicarbonitrile) (Fig. 3C) is a selective inhibitor of USP8, identified in a screen for compounds to overcome resistance to EGFR inhibitors for the treatment of NSCLC [114]. EGFR commonly acquires mutations after treatment with this class of inhibitors [115], spurring the research for alternative methods of targeting this pathway. USP8i was shown to overcome resistance to EGFR inhibitor gefitinib and reduced the cell surface expression levels of EGFR and other receptor tyrosine kinases (RTK) ERBB2, ERBB3, and MET.

### Betulinic acid

Betulinic acid (BA, (3β)-3-hydroxy-lup-20(29)-en-28-oic acid) is a natural compound of plant origin with pro-apoptotic effects and high specificity to cancer cells [116, 117]. This high specificity has caused widespread interest in the compound, with over 100 publications on the subject listed as published in 2016 on PubMed. Yet, neither the exact mechanism by which BA causes its apoptotic effect nor its specificity is fully understood. BA was determined to be a promiscuous DUB inhibitor in the context of prostate cancer, where it induced apoptosis by release of mitochondrial proteins and caused downregulation of angiogenic markers *in vitro* [118]. Interestingly, a recent study of BA suggested an entirely novel pathway in which BA induces apoptosis. In this newly suggested mechanism inhibition of *de novo* fatty acid synthesis is the primary mode of action of the compound [119]. The specificity of BA is explained by the unique reliance of *de novo* synthesis of fatty acids by cancer cells, rather than the uptake from blood as is the case for healthy cells [120]. BA is postulated to cause accumulation of mitochondrial lipid, cardiolipin, which causes structural failure of the mitochondria and consequently the activation of the mitochondria apoptotic pathway. As of yet, it is unclear whether this effect of BA is mutually exclusive with its role as a DUB inhibitor or whether two distinct mechanisms are at play.

### Capzimin

Capzimin (quinolone-8-thiol (8TQ)) selectively inhibits the proteasomal DUB Rpn11/POH1 through direct binding to the catalytic Zn^2+^ ion in POH1’s active site. Recently identified, this compound represents a first-in-class inhibitor selective for POH1 capable of inducing polyubiquitinated protein accumulation and cell death [121, 122]. The compound has so-far been screened against the NCI 60 panel of cell lines and has shown to be effective in leukemia cell lines as well as cell lines derived from solid tumors [121]. Interestingly, capzimin was found to remain efficacious against cells with acquired resistance to bortezomib. Together with the finding that POH1 inhibition induced a distinctly different change in the ubiquitination profile from bortezomib, this compound may have potential as a treatment modality in patients previously treated with bortezomib.

## Ubiquitinating enzyme inhibitors

Further upstream from both the 26S and non-proteasomal DUBs, the ubiquitinating enzymes present an additional target for therapy. Small-molecule inhibitors targeting ubiquitinating enzymes are currently being investigated for their anti-cancer effect and are briefly summarized here.

### MLN4924

MLN4924 (sulfamic acid, [(1S,2S,4R)-4-[4-[[(1S)-2,3-dihydro-1H-inden-1-yl]amino]-7H-pyrrolo[2,3-d]pyrimidin-7-yl]-2-hydroxycyclopentyl]methyl ester) is a first-in-class compound that mimics E1 in the ubiquitination process. However, MLN4924 is incompatible with the following enzymatic reactions, thereby blocking the ubiquitination process by *de facto* inhibiting E1 [123, 124]. In pre-clinical studies, MLN4924 showed potent anti-tumor effects at well-tolerated doses in xenograft models through stabilization of cell cycle regulatory substrates, causing deregulation of DNA synthesis in S phase and apoptosis induction [124]. This novel compound is in early phases of clinical trials for various malignancies and is showing promising initial toxicity profiles in MM and lymphoma [125], melanoma [126], and advanced solid tumors [127].

### PYZD-4409 and PYR-41

PYZD-4409 (1-(3-chloro-4-fluorophenyl)-4-[(5-nitro-2-furanyl)methylene]-3,5-pyrazolidinedione) and PYR-41 (ethyl 4-[(4Z)-4-[(5-nitrofuran-2-yl)methylidene]-3,5-dioxopyrazolidin-1-yl]benzoate) are pyrazone-derived compounds that inhibit E1 [128–130] . PYR-41 shows promising effects on the NF-κB pathway and stabilization of p53 [129]. The compound may have further clinical validity through stabilization of ionotropic testosterone receptor TRPM8 in prostate cancer, implicated to sensitize this type of cancer to apoptosis [131]. PYZD-4409 shows promising anti-tumor activity in primary leukemia patient cells as well as *in vivo* leukemia models [128]. Accumulation of p53 is observed in these models; however, the authors postulate that accumulation of misfolded proteins by inhibition of the UPS and consequential ER stress, rather than the regulation of specific factors, is the primary driver of apoptosis.

### E3 ligase inhibitors

The study of E3 ligase inhibitors has largely focused on antagonizing the binding of E3 ligases to specific substrates [18]. Inhibition of E3 ligases hinges on competing with the binding of specific protein substrates to the E3 ligase, making this treatment potentially highly specific for the intended substrate but also a challenging mechanism to design drugs for. The HDM2-p53 interaction is so far the most promising E3-substrate interaction, for which some compounds have been suggested. Cis-imidazolines, benzodiazepinediones, spiro-oxindoles, and oxindoles are examples of classes of compounds under investigation as stabilizers of p53 through E3 antagonism. To date, p53 stabilization and subsequent induction of apoptosis have been determined in various studies showing both *in vitro* and *in vivo* efficacy, giving strong clinical validity to this class of compounds [131–137]. Importantly, cis-imidazoline Nutlin-3 treatment remained effective in the context of mutated p53 in combination treatment with TrxR inhibitor dasatinib. Nutlin-3 has further shown to inhibit angiogenic factors by p53-dependent inhibition of HIF-1α and p53-independent inhibition of HIF-1 [138].

### Conclusion and future perspective

The UPS is a complex system, and not all of its constituents have been fully assessed. What is well understood is that the UPS is crucially involved in nearly all cellular processes. Considering the importance of the UPS in cancer formation and its validity as a cancer treatment, there is great potential for progress yet to be made in this field. Inhibition of the 20S is clinically established and remains a promising field of study. There is currently a high volume of ongoing clinical trials testing established and new inhibitors of the 20S. These trials aim to find novel 20S inhibitors with increased efficacy and decreased toxicity, as well as discovery of synergistic combination therapies and application in a broader range of cancer types of established inhibitors. Pre-clinically, the unraveling of the role of the vast range of ubiquitinating and deubiquitinating proteins is certain to provide additional targets for treatment, broadening our toolkit for targeting the stabilization and degradation of specific proteins as well as overcoming resistance to 20S inhibitors. Inhibition of the DUBs associated with the proteasome is a direct alternative to the proteasome inhibition facilitated by 20S inhibitors, causing accumulation of poly-Ub tagged proteins and ER stress. Ours and various other labs are working intensively on elucidating the effect of proteasomal DUB inhibition and to facilitate its advancement into clinical practice. New small-molecules with optimized DUB inhibitory effects and pharmacodynamics are being investigated for this goal. Overall, advancement of targeting the UPS in cancer treatment is of significant clinical importance, and we expect to see an increased role for UPS-based treatment in the clinic in the near future.
